# Pointing Treatments Are Task Relevant: A Visuomotor Priming Study

**DOI:** 10.1371/journal.pone.0096154

**Published:** 2014-04-28

**Authors:** Kevin Roche, Hanna Chainay

**Affiliations:** 1 Laboratoire d’Etude des Mécanismes Cognitifs, Université Lyon 2, Bron, France; Centre de Neuroscience Cognitive, France

## Abstract

The present study focused on priming effects on pointing with everyday objects. In a set of four experiments, a visuomotor priming paradigm was used to investigate the nature of visuomotor processing (automatic versus task relevant). By manipulating congruency of orientation and location we found that location congruency facilitates the initiation time of pointing whereas orientation congruency does not. We provide evidence to show that motor planning is influenced by the goal of the action, and that how visual information is processed and held in memory depends on the task relevance. These data are consistent with the proposed interaction between visuomotor and higher processes during the planning and execution of actions such as pointing.

## Introduction

Every day we see different objects in our environment, and often we interact with them. Many different actions can be performed on any given object, but our present study focuses on the planning of pointing (for the sake of touching an object rather than for grasping it) [1]–[3]. We investigated what kind of information is momentarily memorized and potentially facilitates planning of pointing. Is it only information relevant for a specific motor task (here pointing) or is it more general information about an action associated with an object? For the purpose of our investigation we used a visuomotor priming paradigm and measured initiation time.

On a general level, every action requires an intention in order to be executed. Pointing and grasping, for example, involve a conscious intention to initiate the action, with the selection of both the appropriate target and action [4]. That implies two kinds of closely linked processing: visual processing of the target, and motor processing to plan and execute the action. It has been suggested that these processes are common in part to pointing and grasping ([5], [6]). However, some distinctions can be made. Pointing (reaching to touch an object) involves transporting the hand and selecting the final location of a single finger. Grasping (reaching to grasp) involves transporting the appropriate hand and shaping it (at very least a thumb/index grip) to suit the target, generally followed by use of the object. To some extent, grasping requires more precise motor control than pointing [5]. Some authors suggested that a specific action involves specific visual processing, because how information is processed depends on the intentions and plans of the actor [7]. One study lends support to this suggestion [1]. In this study, participants were given the task of looking and pointing at the target or looking and grasping it. The authors found that the first ocular saccade made by participants was affected by the action goal but not by the action irrelevant information, the color of the target. The first saccade tended to go to distractors having the same orientation as the target only when a grasping response was required. This suggests that object orientation is more pertinent for the selection process when a grasp response, rather than a point response, is required. Using a similar approach, some authors [8] also found that a grasping response, but not a pointing one, was influenced by a change in the object's orientation.

However, the aforementioned experiments used bars, two- or three-dimensional shapes, or pictures to investigate visual processing of the action target. One may ask whether the same processing occurs when the target is a real everyday object with a specific function. It is possible that the presentation of real objects, closer to everyday actions and lifelong experiences, can induce stronger or different effects. In one recent study [9], participants had to grasp, touch with a closed fist (similar to pointing), or grasp with a magnetic implement a glass placed either upright or upside-down. The authors found shorter ITs for the regular grasping task, when the glass was placed upright as opposed to upside-down. This effect disappeared when participants touched the glass with their fist, only to reappear when they grasped it with the magnetic implement. Here again, the results suggest an object does not activate motor components automatically but with respect to the purpose of the action and the actors' possibilities. One study [10] lends extra support to the proposal that the variations in how different properties of visual stimuli are treated depend on the selected action. This study involved participants watching video clips of grasping or pointing actions prior to the task of detecting a target that differed in size or location. Watching grasping facilitated responses to a size-defined target, whereas watching pointing facilitated responses to a location-defined target. These results suggest that activating a grasp action made size relevant, whereas watching a pointing action made the target location relevant.

However, it was suggested that in addition to task-relevant processing, grasping processing can be activated automatically because it is the most common action with a tool [11]. In this way, non-relevant information can be processed because it is more likely to be useful in the majority of situations. In fact, they did not observe differences in initiation time (IT) between grasping and pointing depending on the nature of distractor (affording grasping or not affording grasping). Some authors [12], [13] have strongly suggested that processing of visual information is automatic and independent of the action and the actor’s intention. Their works strongly support the hypothesis of automatic activation of irrelevant motor components, *inter alia* orientation [12], [14], size, and semantic properties activated by the noun [15].

To sum up, some studies suggest that depending on the action (pointing, grasping), only pertinent information is processed. It would seem that grasping requires information about size and orientation, whereas pointing requires information about the target's location. Based on the aforementioned studies (e.g. [2], [8]) and the proposition that what kind of information is processed depends on the intentions and plans of the person concerned [7], different predictions can be made about visuomotor priming effects on pointing versus grasping tasks. Based on the proposition that visuomotor processing is task-irrelevant, however, similar effects may be expected on grasping and pointing tasks in a priming paradigm.

In our previous study [3], which focused on grasping initiation, we found facilitating effects of priming on visually guided grasping, but only when the target-object was preceded by an identical prime-object. In addition, priming effects were greater when the orientation between prime and target was congruent. In keeping with other studies, our results indicate that grasping is sensitive to a change in orientation [1], [10], [8]. Interestingly, our priming effects occurred only when the prime was a potential target. This is compatible with the assumption that motor components are evoked in relation to the treatments relevant to the task and depending on the actor’s possibilities of performing a given action [12], [9]. Thus, visuomotor processing is not evoked automatically but depends on a number of different factors such as task (or action) relevance and the actor's intention.

In the present study we examined the automaticity of visuomotor processing by using the pointing task. We were interested in the extent to which information relevant to grasping influences a pointing task, or, more precisely, we were keen to establish whether information about target orientation also influences the planning of a pointing task, either explicitly or implicitly. As in our study involving a grasping task [3], we used a visuomotor priming paradigm with real objects as prime and target. Four experiments were conducted in which the participants had to point to an object and lightly touch it (here, the "pointing task"). The difference between these experiments is at the level of the instructions and, consequently, to which part of the object participants are asked to point.

**Figure 1:**
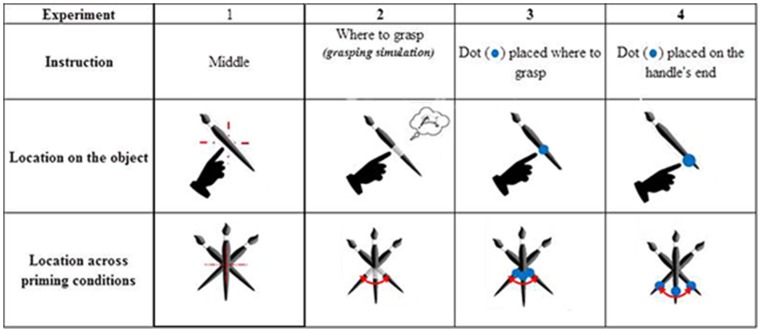
Representation of the location and orientation changes across the priming conditions in the different experiments conducted as part of this study.

In Experiment 1, we asked participants to point to the middle of the object. This part of the object remained constant between the prime and target, unlike the orientation of the object’s handle, which could be in the same or opposite direction. Thus, in Experiment 1, there was always priming congruency for the intrinsic (relative to the object) and extrinsic (relative to the space) location but not always for orientation (see Figure 1).

If visuomotor processing depends on the action selected, and object orientation is irrelevant for the pointing task, we expected to find a general priming effect due to the fact that the object was identical as prime and target (same function and perceptual properties), and not due to orientation congruency (different orientation of prime and target). On the other hand, if visuomotor processing is evoked automatically and independently of the action, priming effects should be found due to the congruency between prime and target of both identity and orientation.

## Experiment 1

### 1 Methods

#### Participants

Twenty-two students (10 men and 12 women) from the University of Lyon 2 took part in the present study. Their mean age was 21.7 years (SD = 3). All of them were right-handed, with normal or corrected-to-normal vision. Before conducting the experiments presented in this study, we obtained the verbal approval from an ethical standpoint of the members of the laboratory. Prior to taking part in the study, the participants had given their written, informed consent in accordance with the Helsinki declaration.

#### Stimuli

The experiment involved 9 everyday objects, all with a handle (knife, hammer, fork, paintbrush, toothbrush, hairbrush, razor, spoon and screwdriver) (see Appendix 1). They were painted black to avoid visual saliency differences between them. A black wooden bar measuring 13 cm long, 3 cm wide and 3 cm high was used as the prime in one of the blocks. In order to avoid acoustic cues as to the nature of the prime, small pieces of felt were attached to the ends of objects, which were in contact with the experimental board.

#### Material

One Dell computer equipped with E-prime2 software (Psychology Software Tools, Inc., USA) was used to run the experiment and record the IT movement. The liquid-crystal goggles (Plato Translucent Technologies, Toronto, Ont.) used to control subjects’ vision were connected to the computer, together with a home-made, 4 cm diameter spherical release button. The primes and objects were placed on a board measuring 40 cm wide and 50 cm long.

#### Procedure

Participants were tested individually. They were positioned facing the experimental board, with their right hand on the release button. The primes and targets were presented on the experimental board one at a time, approximately 45 cm from the participant and with their handles turned towards the participant, at 45° to either the right or left of the participant’s midline, except in the case of the neutral prime which was oriented at 0° to the participant's midline. The objects were rotated to the left and right in such a way that their middle point remained on the participant’s midline.

**Figure 2:**
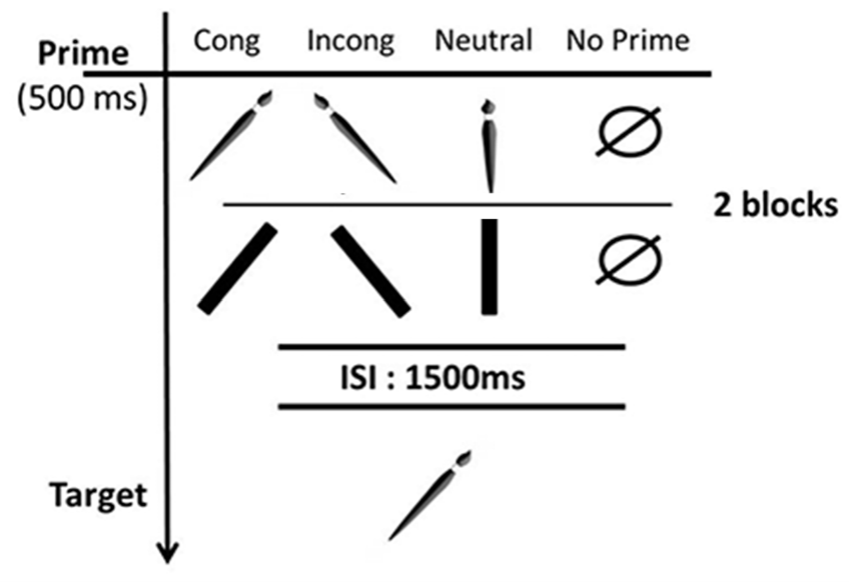
Schematic representation of the different priming conditions used in the experiments conducted as part of this study.

Each participant performed 10 training trials followed by 2 blocks of 72 trials each: one with the object as prime (OP) and the other with the bar as prime (BP). The order in which the blocks were presented was counterbalanced across participants. Four priming conditions were used in each block: (1) congruent orientation between prime and target; (2) incongruent orientation between prime and target; (3) neutral prime, i.e., on the participant's sagittal axis; and (4) no prime (see Figure 2). Each target was presented once in each priming condition. In addition, in each priming condition, the target was turned once to the left and once to the right of the participant. Thus, each object was presented 8 times. The trials in both blocks (OP and BP) were divided into 3 mini-blocks, counterbalanced across participants.

All the trials started with a “beep” to remind the participant to place his/her hand on the release button. Simultaneously, the goggles became opaque for 1500 ms, during which time a prime was placed on the experimental board. The goggles then became transparent for 500 ms so that the prime was visible (or nothing in the no-prime condition), before turning opaque again for a further 1500 ms. During the ISI the experimenter replaced the prime on the experiment board with the target. At the end of the ISI the goggles became transparent again, and a simultaneous “go” signal indicated to the participant that he/she should point to the middle of the target. The next trial then started with a “beep”. The participants were told to point to the object, as quickly as possible. They were given 2000 ms to do so. At no point during the experiment did the experimenter evoke the possibility of grasping or using the object. When the prime and the target had the same orientation and also in the no-prime condition, the experimenter always made noise to prevent the participants from anticipating the orientation of the upcoming target in the first case and from detecting the no-prime condition in the second case. The same applied to all experiments presented in this study.

In our previous study using a similar paradigm [3], we observed an effect of congruent priming on object grasping. To be sure that this effect was not due to the fact that participants anticipated seeing the target oriented in the same direction as the prime (because only in this condition was there no noise due to items being moved) we ran control trials where participants heard a noise during ISI. Although participants could not anticipate congruency from the absence of any noise caused by changing the prime, we still observed effects of congruent priming on grasping. Thus we have shown that simple methodological precautions such as attaching small pieces of felt to the ends of objects and making noise during ISI are sufficient to prevent participants from anticipating the orientation of the upcoming target.

#### Analyses

We measured IT, which was the time that elapsed between the “go” signal and when participants removed their hand from the release button. Preliminary analyses were conducted to check for normality (Shapiro-Wilk’s test) and sphericity (Mauchley’s test), with no violations found. A repeated measure ANOVA was performed, with Prime Identity (OP vs BP), and Prime (Congruent vs. Incongruent vs. Neutral vs. No Prime) as factors. Given that we tested specific hypotheses, planned comparisons were performed, which, unlike post-hoc tests, are in no need of adjustment. In light of criticism in the literature leveled at Bonferroni and other corrections [16], the analyses were performed without adjustment. The same applies to all data presented in this article. Therefore, a significance level of *a = *.05 was used for all statistical analyses. For control purposes we checked for a possible Target Orientation effect (Right Side vs. Left Side) but found no significant difference (F(1,21) = 0.004, p = .94, η^2^ = .0002). Nor did we observe any overt spatial errors concerning the pointing location. Participants always pointed to the expected location.

### 2 Results

The ANOVA showed a significant effect of Prime (F(3,63) = 4.18, p<.01, η^2^ = .16) but not Prime Identity (F(1,21) = .95, p = .33, η^2^ = .04).

**Figure 3:**
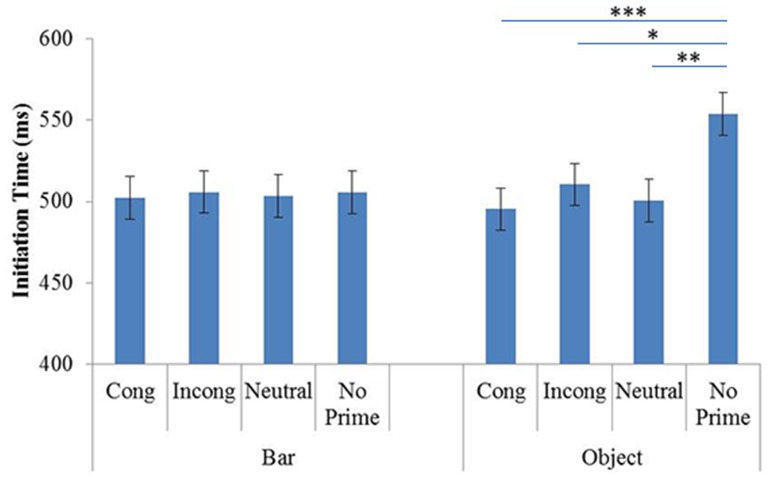
Mean ITs in Experiment 1, as a function of Prime Identity (Object and Bar) and Primes (Cong: Congruent; Incong: Incongruent; Neutral and No prime). Error Bars represent 95% within-subjects confidence intervals (Loftus & Masson, 1994).

The interaction between Prime and Prime Identity was significant (F(3,63) = 5.06, p<.01, η^2^ = .19). The planned comparisons showed no significant effects of priming on ITs in the BP block (see Figure 3). However, such effects were observed in the OP block, in that participants took longer to initiate pointing in the no-prime condition (mean = 553 msec) than in the prime conditions: congruent (mean = 495 msec; p<.001), incongruent (mean = 510 msec; p<.05) and neutral (mean = 500 msec; p<.01). The other comparisons failed to reveal any significant differences, in particular the congruent vs. incongruent condition (p = .36). To investigate this point further we performed an additional Bayesian analysis. To obtain more arguments for the absence of significant differences between the congruent and incongruent conditions, we performed an additional Bayesian analysis on the data concerning these prime conditions in both OP and BP blocks. This allowed us to test which hypothesis, absence of priming (null hypothesis) or presence of priming (alternative hypothesis) is more strongly supported by our data. We used Masson’s method (2011). According to this analysis our data lend more support to the null hypothesis (absence of priming effect). The *a posteriori* probability of the null hypothesis (p_BIC_(H_O_|D) = 0.94) is greater than that of the alternative hypothesis (p_BIC_(H_1_| D) = 0.06). The result of this analysis is positive (almost strong) evidence that the IT in Experiment 1 did not significantly differ depending on stimuli orientation (congruent versus incongruent).

As regards the differences in pointing initiation between the OP and BP blocks, the planned comparisons showed that participants were faster in the BP block than in the OP block in the no-prime condition (p<.001).

### 3 Discussion

The results showed a general priming effect only in the OP block, where the perceptual and functional target properties were known well in advance because the prime and target were the same object. More specifically, ITs were shorter when a prime was presented (independently of orientation congruency) than in the no-prime condition. In addition, the no-prime condition was the only condition where there was a difference between the OP and BP, with longer ITs in the OP block. We found the same effect in our previous study, which focused on priming of grasping [3]. To account for this result we proposed that seeing an object can activate its representation based on allocentric information and its functional identity, whereas seeing a bar does not activate precise representation [17], and that this representation is important for priming target grasping. The data from the present study suggest that it can also be true for the pointing action.

The priming effect observed in the OP block can also be explained in a different way, as being due to a perturbation of the participant's expectation to see the prime. The fact that significant differences between the with-prime conditions (irrespective of orientation) and the no-prime condition were lacking in the BP block but were present in the OP block may be due to the possibility, or not, of forming such expectations. The notion of expectation is bound up closely with the selection of relevant information [7], insofar as processes in charge of selecting information can pre-activate visuomotor processing based on the expectation of a situation involving a specific motor task [10]. From this point of view, participants were unable to form any expectation during the BP block because in this condition the prime never yielded information about where the participants should be pointing. By contrast, in the OP block primes always shared visual information with the target and therefore always yielded information relevant to the task at hand. When such information was lacking (no-prime condition), the IT was longer because the likelihood “scenario” was shattered, and participants needed to adjust to a rare situation.

Concerning our hypotheses about automatic or task-relevant visual processing, our results are inconsistent with the hypothesis that grasping is automatically activated whenever tools are presented, independently of the task [18], and has facilitating effects for action performance. According to this hypothesis and the suggestion that grasping an object involves attending to its orientation [1], [8], the same priming effect of orientation congruency should be expected on pointing and grasping. Contrary to that expectation, however, in the present experiment no such effect on pointing was observed when the goal was to point to the middle of the target.

Taken together, the present data and the observation that grasping an object requires information about its orientation [1], [8], [3] are evidence that orientation processing is rather task-specific, irrelevant for pointing and relevant for grasping. This is consistent with the idea that the processing of visual information is not automatic but determined by the selected action [7], [10].

However, we cannot rule out the possibility that the lack of a location congruency effect is due to motor preservation between trials [19] insofar as the middle of the targets remained constant across trials (the same extrinsic and intrinsic location). This possibility is consistent with our previous explanation suggesting orientation processing is irrelevant for a pointing task.

In Experiment 2 we investigated whether irrelevant information can induce an effect depending on the task. More specifically, does an explicit instruction about grasping in a pointing task induce orientation treatment that influences the planning of pointing?

## Experiment 2

In Experiment 1, participants were asked to point to the middle of an object, and no priming effect was observed. We interpreted these results as evidence that attending to an object's orientation is determined by the selected action. Because this information is relevant for grasping but not pointing, it is processed only when grasping is required. To investigate this suggestion further, we conducted Experiment 2 where the selection of the final pointing location was determined by mental simulation of grasping. The pointing spot in this case was not always in the same extrinsic and intrinsic location across trials. However, in a trial the intrinsic location of the pointing spot was the same between prime and target, and the extrinsic location was either the same or different. Because a priming effect of orientation congruency was observed with the grasping task, we expected to observe it with the pointing task as well, insofar as grasping processing was activated during pointing. To activate this processing, we asked participants to point to the part of the target “where you prefer to grasp it”. We assumed that to execute this task, participants had to simulate grasping before choosing the final pointing spot on the object and initiating the movement. This assumption was based on the suggestion that seeing, simulating and executing an action bear strong similarities with each other, and thus, share the same processing [20], [21]. Consequently, we expected to trigger grasping processing that was strong enough to interfere with pointing task processing.

We expected to find a priming effect of orientation congruency if the simulation of grasping movement was sufficient for attending to an object’s orientation even if orientation was irrelevant for performing a pointing movement. Conversely, if simulation of grasping was insufficient for attending to an object’s orientation, we expected to replicate the lack of orientation congruency effect found in Experiment 1.

### 1 Method

#### Participants

Twenty-five students (11 men and 14 women) from the University of Lyon 2 took part in the present study. Their mean age was 22 years (SD = 4). All were right-handed, with normal or corrected-to-normal vision. Prior to their participation in the experiment they had given their written, informed consent.

The same material, procedure, stimuli and analyses were used as in Experiment 1, except for the instructions given to the participants, who were told to point to the best part of the object for grasping it successfully, precisely at the spot between the hypothetical grasp positions of thumb and index. We observed no overt spatial errors in this task. We checked for a possible Target Orientation effect (Right Side vs. Left Side) but found no significant difference (F(1,24) = 1.51 p = .23, η^2^ = 0.05).

### 2 Results

The ANOVA showed a significant effect of Prime (F(3,72) = 7.04, p<.001, η^2^ = .21) but not Prime Identity (F(1,24) = .51, p = .47, η^2^ = .51).

**Figure 4:**
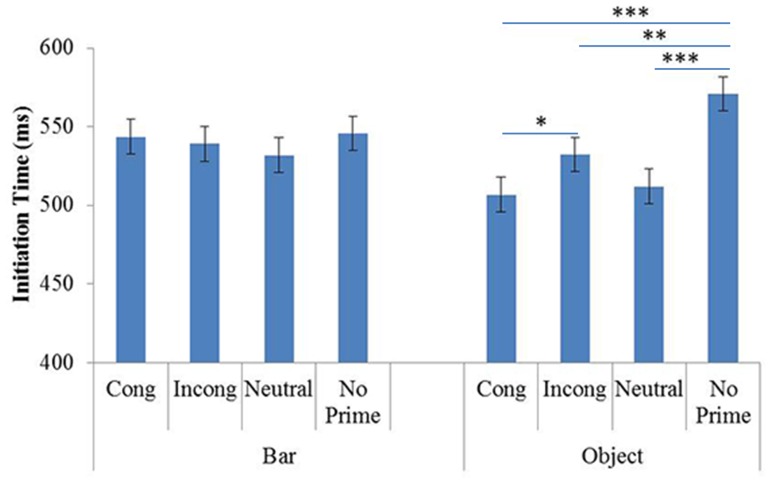
Mean ITs in Experiment 2, as a function of Prime Identity (Object and Bar) and Primes (Cong: Congruent; Incong: Incongruent; Neutral and No prime). Error Bars represent 95% within-subjects confidence intervals.

The Prime and Prime Identity interaction was significant (F(3,72) = 7.74, p<.001, η^2^ = .23). The planned comparisons did not reveal any significant effect of priming on ITs in the BP block (see Figure 4). Such effects were observed only in the OP block, where participants took less time to initiate pointing in the congruent condition (mean = 506 ms) than in the incongruent condition (mean = 532 ms; p<.05). In addition, the no-prime condition (mean = 570 ms) induced longer ITs than the other, with-prime conditions: congruent (p<.001), incongruent (p<.01) and neutral (mean = 512 ms; p<.001). The other comparisons failed to show any significant differences. Concerning the differences in ITs between the OP and BP blocks, the planned comparisons showed that participants were faster in the OP block than in the BP block for the congruent condition (p<.05).

### 3 Discussion

As in Experiment 1, effects of priming were observed only in the OP block. Participants initiated pointing faster when the prime preceded the target than in the no-prime condition. More interestingly, in the OP block, participants initiated pointing faster in the orientation congruent condition than in the incongruent condition. This difference is probably due to the fact that, unlike in Experiment 1, in the present experiment participants had to activate grasp related information, as they were asked to point to the most appropriate spot for grasping a given object. However, the facilitator congruency effect can also be interpreted in terms other than that of attending to an object’s orientation. Given that pointing has been shown to be sensitive to changes in location [10], [22] it is possible that the observed effect of congruency was the result of a constant location (in the congruent condition), which induced easier pointing planning. Two further experiments were run to attempt to distinguish between these two possible effects, congruency of orientation and congruency of location.

Experiment 3 was run to find out whether the effects observed in Experiment 2 were due to intentional simulation of grasping. In other words, we set out to check whether the congruency effects were due to attending to task-irrelevant object orientation, induced by intentional grasping simulation, or a congruency of location. Before running this experiment, we established where individuals generally tend to grasp an object. We marked the precise spot, and the instruction given to participants was simply to point to the mark. No mention was made of grasping, and participants were not supposed to simulate grasping intentionally. Therefore, in Experiment 3 we predicted that if the point location is the same as the grasp location it is sufficient to activate grasping processing implicitly and automatically and to induce an orientation congruency effect [10], [11] on the pointing task.

In Experiment 4, the idea was to distinguish between effects induced by simulation of grasping and attending to task-irrelevant orientation, and those induced by task-relevant location processing. Thus, in this experiment participants were asked to point to the precise spot, which was not where individuals generally tend to grasp an object. As in Experiment 3, no mention was made of grasping. If the effects of priming observed in Experiments 2 and 3 were the result of grasping simulation and attending to orientation, due in the former to intentional evocation of grasping and in the latter to its implicit evocation via the location of the pointing spot (which was grasping-related), no priming effects should be observed in Experiment 4.

## Experiment 3

The main aim of this experiment was to examine whether priming effects observed in Experiment 2 were due to the intentional simulation of grasping and to orientation congruency. If the orientation congruency effect observed in Experiment 2 were not to be found in Experiment 3 the indication would be that the effects observed in Experiment 2 are actually due to orientation congruency processing.

### 1 Methods

#### Participants

Twenty students (8 men and 12 women) from the University of Lyon 2 took part in the present study. Their mean age was 20.7 years (SD = 2). All were right-handed, with normal or corrected-to-normal vision. Prior to their participation in the experiment they had given their written, informed consent.

The material, procedure, stimuli and analyses were the same as in previous experiments, except for the instructions given to the participants and a marking placed on the stimuli used for both the prime and the target. To identify the position of the marking, we asked 10 volunteers to point randomly to the best grasping location on 9 objects used as stimuli in the two target orientations, 45° to the right or left of the participants' midline. We calculated the mean location in order to keep a single location per object. We marked this location on each object (including the bar as the prime) with a red marking (diameter: 8 mm). The locations clearly differed between objects and in terms of their respective middle, but they did not vary according to the orientation of the target. Thus, as in Experiment 2, in a trial, the intrinsic location of the pointing spot did not differ between prime and target, but the extrinsic location did, depending on the orientation congruency between the two stimuli. Participants were instructed to point to the precise marking on the target. We observed no overt spatial errors in this task. Participants always pointed correctly to the marked spot. We checked for a possible Target Orientation effect (Right Side vs. Left Side) but found no significant difference (F(1,19) = 0.51 p = .48, η^2^ = 0.02).

### 2 Results

The ANOVA showed a significant effect of Prime (F(3,57) = 15, p<.001, η^2^ = .45) but not of Prime Identity (F(1,19) = .004, p = .94, η^2^ = .0002).

**Figure 5:**
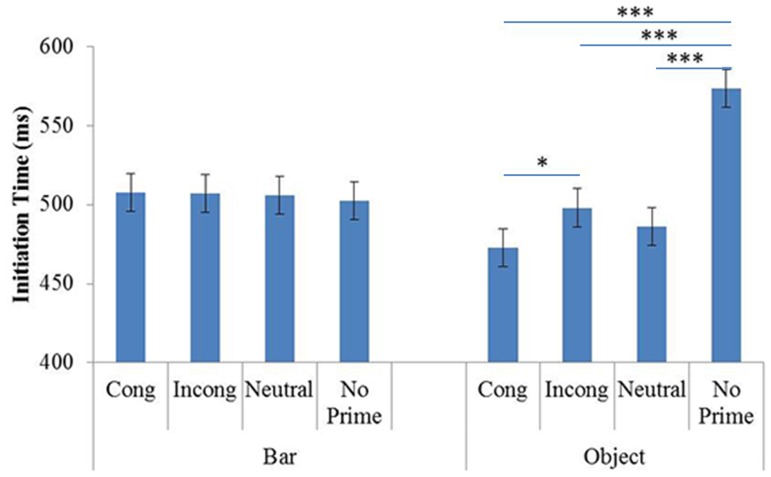
Mean ITs in Experiment 3, as a function of Prime Identity (Object and Bar) and Primes (Cong: Congruent; Incong: Incongruent; Neutral and No prime). Error Bars represent 95% within-subjects confidence intervals.

The interaction between Prime and Prime Identity (F(3,57) = 28, p<.001, η^2^ = .60) was significant. The planned comparisons showed no significant effect of priming on ITs in the BP block (see Figure 5). Such effects were observed only in the OP block, with participants taking less time to initiate pointing in the congruent condition (mean = 472 ms) than in the incongruent condition (mean = 497 ms; p<.05). In addition, the no-prime condition (mean = 573 ms) induced longer ITs than the other, with-prime conditions: congruent (p<.001), incongruent (p<.001) and neutral (mean = 486 ms; p<.001). The other comparisons revealed no significant differences. Concerning the differences in grasping initiation between the OP and BP blocks, the planned comparisons showed that participants were significantly faster in the BP block than in the OP block in the no-prime condition (p<.05).

The data from this experiment will be discussed together with the data from Experiment 4.

## Experiment 4

With this last experiment we checked whether the congruency effects on pointing were induced by attending to task-irrelevant object orientation, or by task-relevant information about location. To that end, the position of the point marking was switched to a location that was irrelevant for correctly grasping objects. Consequently, if the congruency effect observed in Experiments 2 and 3 persisted in this Experiment, it would be due to location congruency, independently of grasp or orientation processing. If no effect of congruency was found, the explanation for the congruency effect observed in Experiments 2 and 3 would be automatically evoked grasp processing and more specifically orientation processing.

### 1 Methods

#### Participants

Twenty students (9 men and 11 women) from the University of Lyon 2 took part in the present study. Their mean age was 20.7 years (SD = 2). All were right-handed, with normal or corrected-to-normal vision. Prior to their participation in the experiment they had given their written, informed consent.

The material, procedure, stimuli and analyses were the same as in the previous experiments, except for the instruction given to the participants and the marking placed on the stimuli used with both the prime and target. Here again, in a trial the intrinsic location of the pointing spot was always the same between prime and target, but the extrinsic location was either the same or different depending on the congruency of orientation between these two stimuli. We deemed that an irrelevant position for correctly grasping objects is approximately 1 cm from the end of the handle. The participants were instructed to point to the exact spot on the target where the marking was. We observed no overt spatial errors in this task. Participants always pointed correctly to the marked spot. We checked for a possible Target Orientation effect (Right Side vs. Left Side) but found no significant difference (F(1,19) = 0.56 p = .46, η^2^ = 0.02).

### 2 Results

The ANOVA revealed a significant effect of Prime (F(3,57) = 58, p<.001, η^2^ = .75) but not of Prime Identity (F(1,19) = .29, p = .94, η^2^ = .01).

**Figure 6:**
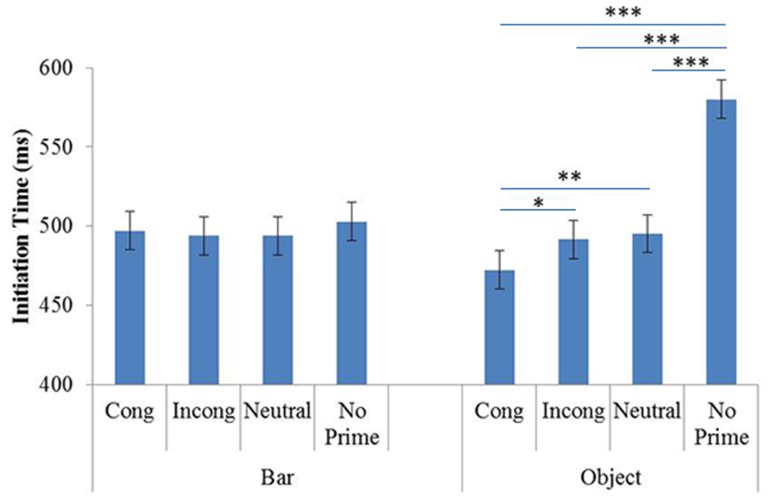
Mean ITs in Experiment 4, as a function of Prime Identity (Object and Bar) and Primes (Cong: Congruent; Incong: Incongruent; Neutral and No prime). Error Bars represent 95% within-subjects confidence intervals.

The interaction between Prime and Prime Identity (F(3,57) = 33, p<.001, η^2^ = .63) was significant. The planned comparisons showed no significant effect of priming on ITs in the BP block (see Figure 6), with such effects observed only in the OP block, where participants took less time to initiate pointing in the congruent condition (mean = 472 ms) than in the incongruent (mean = 491 ms; p<.01) or neutral condition (mean = 495 ms; p<.001). In addition, the no-prime condition (mean = 580 ms) induced longer ITs than the other with-prime conditions: congruent (p<.001), incongruent (p<.001) and neutral (p<.001). The other comparisons failed to reveal any significant differences. Concerning the differences in grasp ITs between the OP and BP blocks, the planned comparisons showed that participants were significantly faster in the BP block than in the OP block in the no-prime condition (p<.01).

To gain a better understanding of the difference between Experiment 1 and the others Experiments (2, 3 and 4) concerning the congruency effect in the OP block (its absence in Experiment 1 and its presence in the other Experiments), we performed an ANOVA with the four Experiments as group factor and congruency (congruent vs. incongruent) as repeated measure factor. The analysis revealed a significant effect of Congruency (F(1,83) = 13.7, p<.001, η^2^ = 0.14). The effect of Experiment (F(1,83) = .6, p = .64, η^2^ = .02) and interaction between Experiment and Congruency (F(3,83) = .2, p = .89, η^2^ = .007) were not significant.

## Discussion of Experiments 3 and 4

The purpose of Experiments 3 and 4 was to distinguish between different potential causes of the priming effects observed in Experiment 2. We found two different priming effects in these three Experiments. Insofar as they were found only in the OP blocks, it can be assumed that the prime induces an effect when it shares information with the target.

Two priming effects were observed in Experiments 3 and 4. First, as in Experiments 1 and 2, the ITs in the no-prime condition were longer than in the prime conditions but only in the OP block. This effect is discussed in the General Discussion section.

Secondly, the congruency effect seen in Experiment 2 remains constant in Experiments 3 and 4. In Experiment 3 we examined in detail whether the congruency effect observed in Experiment 2 is specific to intentional simulation of grasping. Given that participants were no longer asked to point to the preferred part of an object for grasping purposes, they had no need to simulate grasping. However, the markings on the primes and targets indicating where to point corresponded to the preferred grasp locations. Despite this modification, the congruency effect persisted in Experiment 3, such that it would appear that the congruency effect observed in Experiment 2 is not specific to intentionally simulated grasping. Thus, insofar as the point location is relevant for grasping it is possible that automatic processing of orientation relevant for grasping may occur and may thus account for the congruency effect observed in Experiment 3. With the data from Experiment 4 it is possible to determine whether the congruency effects observed in the pointing task in Experiment 3 are dependent on attending to object orientation relevant for grasping. We replicated the congruency effect with instructions to point to a marking at a location that was irrelevant for efficient grasping (close to the end of the handle).

According to the proposition that attending to an object's orientation is grasping-task relevant [1], and grasping is not automatically evoked (Experiment 1 and also [9]; [10]), it would seem that the congruency effect observed in Experiment 4 was due more to location processing *per se* than to orientation processing. It is consistent with the proposition that pointing processes are sensitive to location changes (or congruency), whereas grasping processes are dependent on orientation changes [1], [3], [8]. We therefore suggest that our congruency priming effect may be explained better in terms of location than orientation congruency.

## General Discussion

In the present study our main interests were two-fold. First, we investigated whether visuomotor processing is automatic or task-relevant by trying to induce activation of irrelevant orientation/grasp processing on a pointing task and also by comparing our findings with our previous study, which focused on a grasping task [3].

First of all, general priming effects (facilitation of pointing by prime regardless of its orientation) were observed in all four experiments, but only in conditions where both prime and target were identical and real objects. These data show it is possible to observe priming effects on pointing initiation with real objects. They do not therefore support the hypothesis that pointing (as well as grasping) is based only on the real-time processing of visual information [4] [23].

In addition, in three of the four experiments, where the extrinsic location of the pointing spot changed, we observed a congruency priming effect on the pointing task that we interpret as due rather to location congruency than orientation congruency. Our data are consistent with previous studies, which showed that pointing requires information about the target's location but not its orientation [1], [2]. In Experiments 2, explicitly, and 3, implicitly, we evoked grasp processing and consequently attending to an object's orientation, and in both experiments we observed faster pointing initiation in the orientation congruent condition between prime and target. One explanation could be that attending to prime orientation, induced by grasping simulation, has influenced initiation of target pointing. We think that as a possible explanation this is overly complicated with respect to data coming from Experiments 1 and 4, where neither the instructions nor the point locations referred to grasping, and thus to objects’ orientation. In these two experiments, changes in orientation always occurred across the different priming conditions. Interestingly, we did not observe any effects of congruency in Experiment 1 where the extrinsic point location remained constant in all priming conditions, but such effects were observed in Experiment 4 where this location differed depending on the priming condition. These data have to be interpreted with caution, because when comparing participants’ performance across experiments, we found no significant difference concerning the congruency effect between Experiment 1 and the others. Instead, we observed a significant main effect of congruency, indicating that in general participants initiated pointing faster in the orientation congruent condition. However, this may be due to the fact that this analysis simply drowned the results from Experiment 1 in the results from other Experiments, as it was the only one where the significant effect of congruency was not observed. An additional Bayesian analysis performed on the data from Experiment 1 showed that these data favour the null hypothesis (absence of congruency effect) over the alternative hypothesis (presence of congruency effect). Thus, it is difficult to interpret the absence of orientation congruency effect observed in Experiment 1 as a mere artefact. Based on these two analyses, we propose that the congruency effect observed in the present study stems from attending to location rather than to an object's orientation, which was irrelevant for the pointing task. Our results lend support to the idea that the information processed varies depending on the action selected [8]. This means that visuomotor processing is non-automatic, task-relevant, and differs according to whether the task is pointing or grasping [3], [8].

Some authors proposed that objects automatically activate visuomotor processing even if it is task irrelevant [12], [14]. Such motor activation was frequently observed in the literature as a compatibility effect between stimulus orientation and responding hand. In one study [12] this compatibility disappeared when participants answered with only one hand. In our previous study which explored the priming effect on grasping [3] and in the present study we asked right-handed participants to use their right hand to perform actions and found no facilitating effect on grasping and pointing at right-oriented targets as opposed to left-oriented targets. Our data support the proposal [24] that the compatibility effect between stimulus orientation and responding hand can be better explained in terms of spatial compatibility as a result of the experimental design rather than in terms of automatic motor activation. Accordingly, the orientation hand compatibility effect in tasks such as pressing a button [12], [14] could be explained by global orientation processing. However, when a task itself requires spatial coding, as it is the case in our studies with the right hand for grasping and pointing at the left- or right-oriented target, this specific coding is dominant and the global orientation processing does not influence action planning. Thus, a situation involving more ecological or complex actions (like grasping and pointing rather than simply pressing a button) seems to activate more specific visuomotor processing to suit the action that is to be performed [9], [24], [25].

However, we cannot discard the possibility that automatic and task-irrelevant processes occur at some levels. Indeed, some studies have revealed a co-occurrence of relevant and irrelevant processing [11], [22]. For example, it has been proposed [22] that different visual processing occurs for different (even irrelevant) tasks, but the necessary motor task optimizes the visuomotor processes and selects the relevant information.

The second priming effect observed throughout all of our experiments was slower ITs in the no-prime condition than in all other prime conditions. We interpreted this effect as being due to a perturbed expectation to see the prime. Given that the prime was presented in the majority of trials, participants could prepare themselves for seeing the prime, and shattering this expectation (no-prime condition) resulted in slower planning of the pointing movement. In the past, we put forward another explanation for this effect, i.e., a functional/allocentric facilitation. It is true that everyday tools can activate a specific representation with allocentric [18] and functional properties [17] and facilitate subsequent action, but it has also been proposed [12] that how object representation is activated depends on the task. Our study shows that a location seen previously influences initiation of a pointing task only when prime and target are the same, real, everyday objects. In the BP block, where the prime was a bar, we did not find any priming effects. However, many of the aforementioned studies found priming/distractor effects with this kind of prime [1], [2], [8], [14]. Therefore, we suggest that the lack of any priming effect in the BP block in our study is due to the fact that the prime has to have the same identity as the target to induce motor processing of egocentric information such as location for pointing and orientation for grasping [3], [14]. To put it another way, the prime became relevant to the action task when it shared information with the target. In addition, the relevance of the prime is closely linked to the agent’s expectation. These two explanations are serious candidates for explaining why the ITs were shorter when the target was seen in the prime conditions than in the no-prime condition in OP block. Further investigations are needed to gain a better understanding of this effect.

In the present study we investigated the effects of orientation and location priming on the initiation time of pointing. Given that the initiation time is thought to reflect action planning and gives no information about action execution, the conclusions stemming from our study are limited to action planning. Different influences could be expected on movement time or kinematics.

More generally, and by way of a conclusion, our study provides some evidence that information processing useful for motor planning and action initiation is influenced by the goal of the action in question. In addition, it seems that information may be held in memory according to its relevance for the goal of the task. Thus, our study supports the hypothesis that there is interaction between visuomotor and both perceptive and cognitive processes for planning an action such as pointing.
